# Effects of row direction and row spacing on maize leaf senescence

**DOI:** 10.1371/journal.pone.0215330

**Published:** 2019-04-18

**Authors:** Chang Tian, Jichang Han, Juan Li, Guo Zhen, Yangyang Liu, Yangjie Lu, Yike Wang, Yang Wang

**Affiliations:** 1 Institute of Land Engineering and Technology, Shaanxi Provincial Land Engineering Construction Group Co., Ltd., Shaanxi, Xi’an, China; 2 Key Laboratory of Degraded and Unused Land Consolidation Engineering, the Ministry of Land and Resources of China, Shaanxi, Xi’an, China; 3 Shaanxi Provincial Land Consolidation Engineering Technology Research Center, Shaanxi, Xi’an, China; 4 Northeast Institute of Geography and Agroecology, CAS, Changchun, China; Huazhong Agriculture University, CHINA

## Abstract

To analyze three row orientations (south-north, east-west, southwestern 20°) and two row spacings (‘65 + 65’, ‘160 + 40’), we investigated the effect of row orientation and planting pattern on photosynthetic performance, physiological and biochemical indicators related to the aging of leaves. Results revealed that during maturity stage, in north-south and east-west, the initial fluorescence (Fo) at ‘65 + 65’ were higher than those under‘160 + 40’; the maximum quantum yield of PS2 photochemistry(Φ_P0_), basal quantum yield of non-photochemical processes in PS2(Φ_N0_)of the lower leaves and photosynthetic rate of the upper and ear leaves under‘160 + 40’were higher than those under‘65 + 65’. The polyphenoloxidase (POD) activities of leaves at different positions under ‘160 + 40’ were higher than that under‘65 + 65’, while the malondialdehyde (MDA) content was lower. The photosynthesis rate, superoxide dismutase (SOD) and catalase (CAT) activity of leaves at different positions under southwestern 20° ‘160 + 40’ were higher than others. Whilst MDA content ‘160 + 40’ were lower. Therefore, in De Hui City, Jilin Province, southwestern 20° ‘160 + 40’ delayed leaf senescence at the late stage of growth of maize, as well as the effect of increasing maize yield was most obvious.

## Introduction

Maize (*Zea mays* L.) has higher yields than rice and wheat. Therefore, maize has spread over China’s most area and become one of China’s major corps. To increase the production of maize, numerous experiments shave been done. Since 1988, Lu et al.[1] and Duan et al.[2] have proved that premature leaf function could largely affect the seed setting rate and further decide the grain yield; Later, Davide[3], Ma and Dwyer[4] demonstrated that premature leaf senescence, reduced green leaf area and shortened photosynthetic time would severely damage the grain yield. So it’s well believed that keeping corps green and prolonging the photosynthetic time can improve the photosynthetic rate after anthesis, and thus significantly increase the grain yield. In 1986, Gentinetta and Brodbeck[5] achieved a significant increase in yield, with an inbred maize which can maintain its greenness well. In 1993, Thomas and Smart[6] showed that the yield would increase when the leaf aging rate decreased. The aforementioned results helped link the leaf aging speed to grain yield. Since seeds and vegetative organs grow simultaneously, how to prolong the leaf function and prevent the prematurely aging have become a major concern[7].

After decades of efforts, researchers found that the corps’ photosynthetic production relied on the canopy’s micro-environment such as light, temperature, humidity and CO_2_[8–11]. Such micro-environment could be enhanced by proper configuration of row direction and row spacing, e.g., row spacing of 70 cm and 50 cm had greater photosynthetic production potential than row spacing of 65 cm and 60 cm[12]. The double-plant corn with wide-narrow row could contribute to slow aging in the middle and late growth period[13]. With row spacing of 15 cm, Chlorophyll degraded slowly, malondialdehyde content decreased, antioxidant system enzyme activity increased. In other words, functional leaf aged more slowly and grain weight per spike increased in flag leaves[14]. Compared to the conventional homogeneous ridge, the wide-narrow row with southwestern 20° led to higher SPAD, non-structural carbohydrates content, SOD and POD activities and lower proline content[15]. Song et al. [16] believed that appropriate row spacing could effectively improve the SOD, POD and CAT activities, so as to maintain the balance of reactive oxygen metabolism, reduce the content of MDA, alleviate the lipid peroxidation of cell membrane, prevent premature aging and ensure the pod yield.

The previous which studies individually on row orientation or row spacing had a greater impact on the senescence of maize leaves. But considering row direction and row spacing comprehensively, the impact on the senescence of maize leaves was less studied. Based on the previous research, this study used field experiments to determine some physical indicators, such as photosynthesis rate and soluble sugar, of different parts of maize during the late growth stage of different row directions and row spacings. In this paper, we utilized the physiological indicators to analyze the effects of row direction and row spacing on maize leaf senescence and discussed the mechanism of planting mode to delay the senescence of maize leaf, which provided a theoretical basis for increasing the yield of maize.

## Materials and methods

### Site

The field experiments were conducted at the Dehui Agricultural Experimental Station, which is located in Songliao Plain in central northeast China (47°27ʹN, 126°55ʹE) and characterized by a mid-temperate continental climate. During the growing seasons, the average annual precipitation was about 520 mm with a frost-free period of 138 days. The average temperature was 4.4°C with a sunshine time of 2,688 hours per year. The soil was a black soil with a pH of 6.6. In the 0–20 cm soil layer, the soil organic matter content was 26.9 g/kg. The total contents of nitrogen, phosphorus, and potassium were 1.20, 1.06 and 16.9 g/kg, respectively. The available contents of nutrientions (nitrogen, phosphorus and potassium) were 119, 18.0 and 111 mg/kg, respectively.

### Experimental design and field management

**Fig 1:**
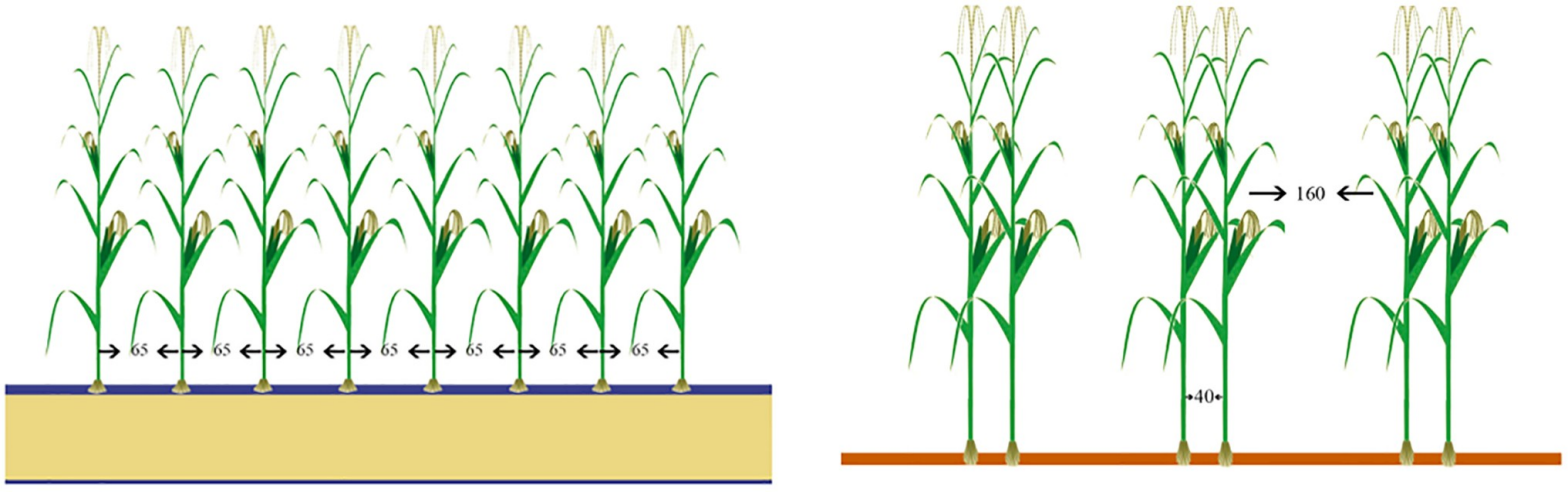
Two different planting patterns(unit:cm). The left picture represents the traditional planting pattern. The other one represents the new planting pattern.

Fields were treated differently using a double-factorial randomized block design with three replications. Each plot was 20 m wide and 30 m long. The experiment involved six treatments: three row orientations (south-north(SN), east-west(EW), southwestern 20°(SW20) and two sets of row spacings (‘65 + 65’, ‘160 + 40’) (Fig 1). Maize (Langyu 99, provided by dandong denghai liangyu seed co. LTD) density is 6.5 plants m^−2^. The fertilizers included240 kg hm^-2^ N, 90 kg hm^-2^ P_2_O_5_ and K_2_O. First, the P fertilizer and 1/3 of the N fertilizer were together applied as the base fertilizer, and then the remaining 2/3 of the N fertilizer was applied at the elongation stage.

When the row direction changed, the shadow length of the corps changed accordingly, which also affected the corps’ sunlight receiving situation. Besides, the shadow length of the crops is also affected by multiple factors, such as solar altitude angle, solar azimuth, solar declination, solar time angle, geographic latitude and crop height. During daytime, the solar radiation received by the groundvaried with the solar altitude. At noon, the solar altitude angle was largest, that is, the corps received the most solar radiation between 9:00 am and 3:00 pm, during which crops could perform strong photosynthesis. Due to the rotation and revolution of the Earth, the diurnal and annual variation of the solar elevation and azimuth would cause changes in the solar elevation angle (h) and solar azimuth (Φ), which directly affected the projection length and direction of the crops on the ground and changed according the solar time angle (ω, obtained by looking up the table) in one week of the Earth’s rotation.

The solar elevation (h) and azimuth (Φ) can be calculated via the below equations:

sinh=sinΦ•sinδ+cosΦ•cosδ•cosω (1)

cosΦ=(sinΦ•sinh-sinδ)/(cosh•cosΦ) (2)

where φ is the geographic latitudeand δ is the declination of the date of the calculation. (Note: Φ is zero degree at positive south, +90° at positive west, -90°at positive east, and ±180° at positive north).

**Fig 2:**
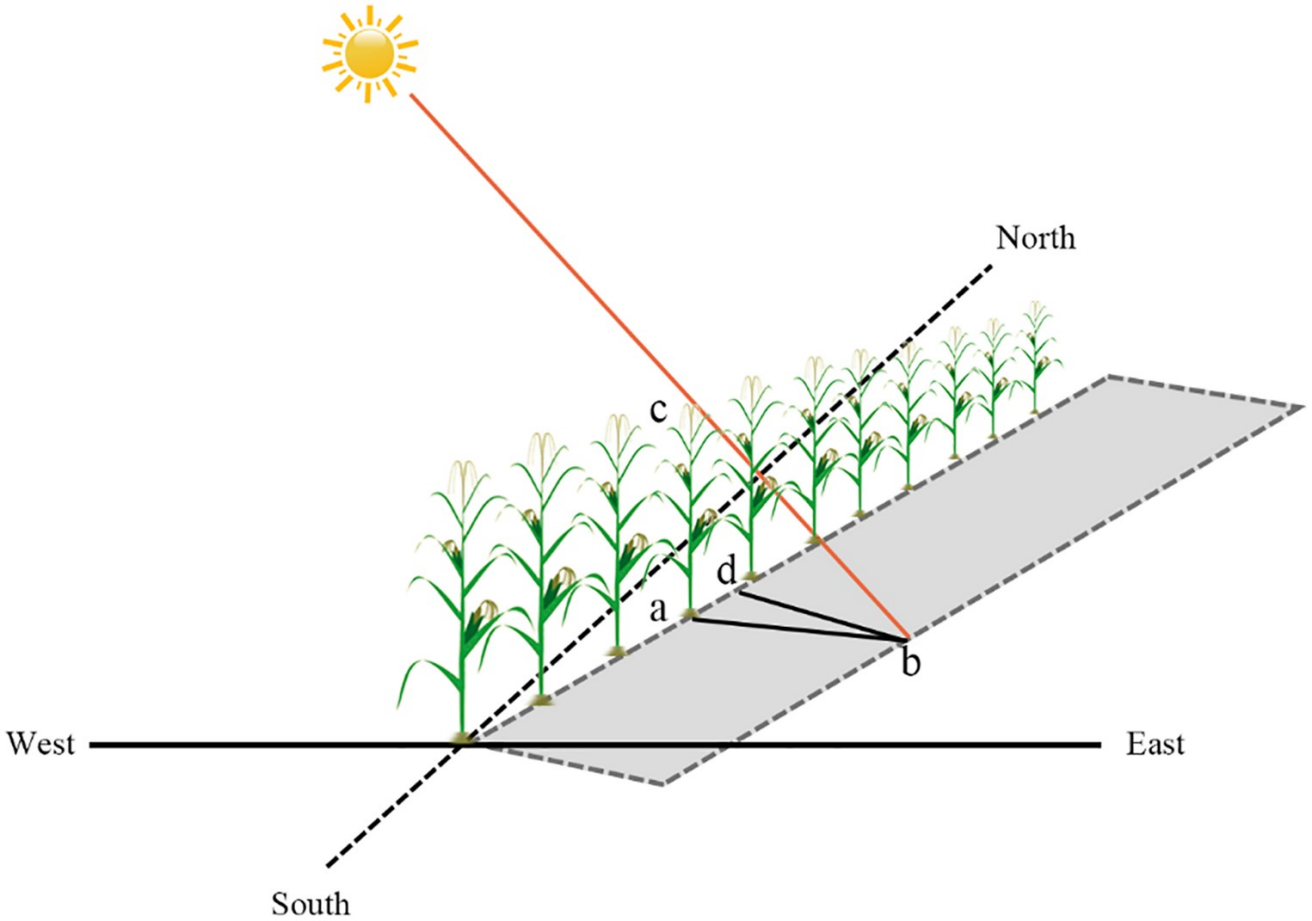
The line projection diagram. ac stands for plant height, ab stands for projection length, and bd stands for row projection width.

By Eqs (1) and (2), we obtained the solar elevation angle (h) and solar azimuth (Φ) of the Dehui Experimental Area in Summer. Fig 2 showed the projection of maize in the fields. We set L as the height of maize, and A as the row direction angle (the angle between row direction and south), then we can obtain the shadow length (YL) and the projection width (TL) as follows:

YL=L•ctgh (3)

TL=YL•sin(Φ±A) (4)

TL=L•ctgh•sin(Φ±A) (5)

(Note: +A and -A are selected to calculate projection width in the morning and afternoon, respectively).

**Fig 3:**
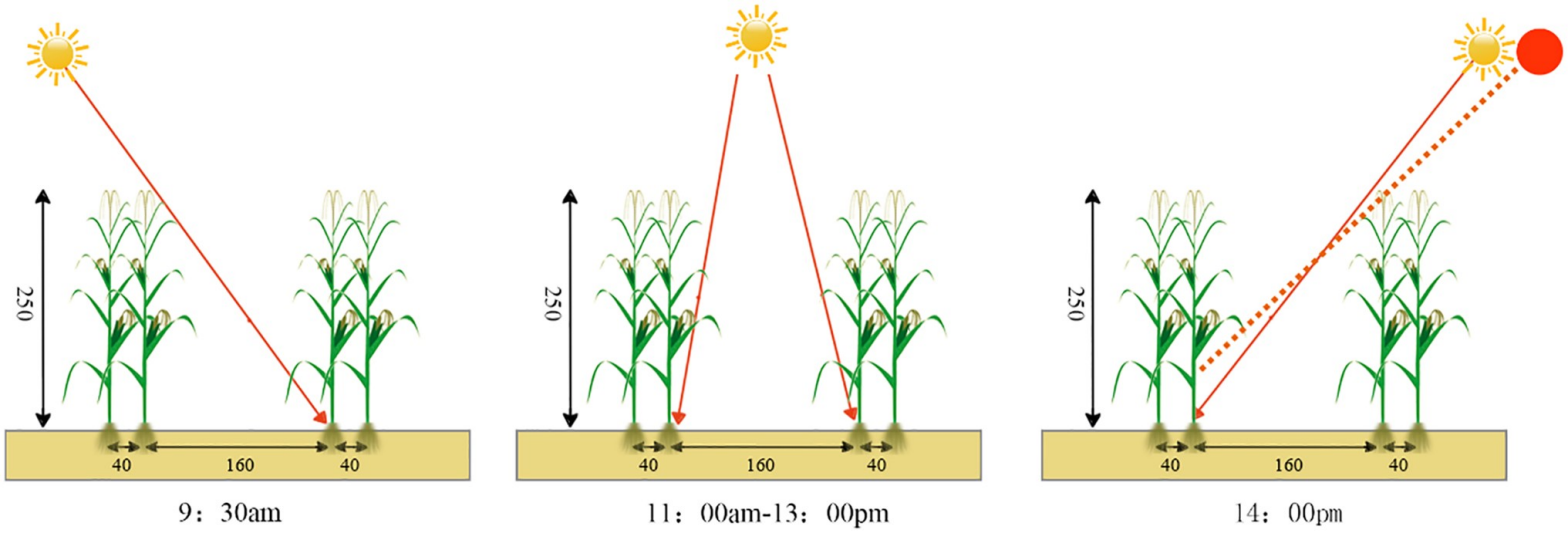
The illumination diagram of the new planting pattern (unit:cm).

**Fig 4:**
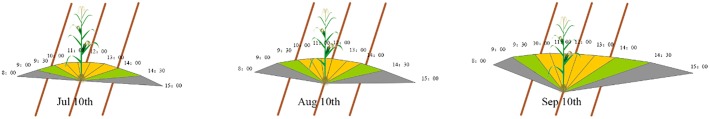
The horizontal shadow distance at different times.

Usually, the plant height of the maize was 2.5m. From July 10th to September 10^th^, A ranged from 0° to 35° at an interval of 5°. We substituted these A values into Eq (3), the obtained heights of maize (YL) ranged between 0.26 to 2.58 m, YL was shortest when A was 20°, whose longest duration was 4.5–5.0 hours during 9:30 am to 2:30 pm. After August 10^th^, the bottom maize leaves (<0.5m height above the ground) naturally aged and lost the ability of photosynthesis. From 10^th^ August to 10^th^ September, we assume that the plant height is 2.0 m, and choose 9:30 am to 2:30 pm as the sampling duration, comprehensive analysis of the horizontal projection length can be shown in Figs 3 and 4. As can be seen in the figures, the horizontal projection length varied from 0–1.6 m, accounting for 78% of the total data. The combination of large ridges and small ridges has been adopted. The large ridges were used to provide ventilation and light transmission environment to plants and the small ridges were used as planting rows. Inspired by the traditional intercropping patterns, we applied uneven distribution to the sides of plants, which increased the light-receiving area and light-receiving duration of the plants.

### Sampling and measurement

In the 2016 field experiments, we applied five-point sampling method, selecting 5 plants in each plot in 5 growth stages: anthesis(7/31), milk stage(8/14), milk-ripe stage(8/29), soft dough stage(9/12), and maturity stage(9/24) (Same as below). The photosynthetic rate and chlorophyll fluorescence parameters of the ninth, twelfth and twentieth leaves (hereinafter referred to as the middle leaves, the upper leaves and the lower leaves) were measured with Li-6400p and MINI-PAM, respectively.

In 2017 field experiments, we also selected five plants in each plot in 5 growth stages, based on five-point sampling method. We avoided the main veins, and instead extracted 1-g samples from the middle of the ninth, twelfth and twentieth leaves on one side. Finally, samples were sent to the laboratory to measure the physiological index.

### Photosynthetic rate

From 10:00 am to 3:00 pm, we used the leaf clip to obtain the middle of the leaf (1 cm away from leaf vein), and let the light intensity be 1000 μmol CO_2_ m^-2^ s^-1^, then we waited for PHOTO reading in the C line to be stable and record it.

### Chlorophyll fluorescence parameters

After 30-min dark adaptation, we first tested Fo with weak light and then measured maximum fluorescence (Fm) with a strong flash (6 000 mol·m^-2^ s^-1^, pulse time 0.7 s). According to Fv = Fm-Fo, Φ_P0_ = Fv/Fm, Φ_N0_ = Fv /Fo, maximum variable Chl fluorescence yield (Fv), Φ_P0_ and Φ_N0_ were calculated.

### Physiological and biochemical indications

All of the analyses were based on the plant physiology experiment technique. Protective enzyme: (i) Protective enzyme extraction: Weigh 0.5 g of fresh leaves and grind them to a homogenate in 5 ml of pre-cooled 0.1 mol/l, pH = 7.8 phosphate buffer into a pre-cooled mortar and pour it into a 10 ml centrifuge tube. Centrifuged at 4000 RPM for 20 minutes, and stored at 0–4 °C[17]. (ii) SOD activity was determined by using nitro-blue tetrazolium (NBT) in the presence of riboflavin[18], POD activity was determined via colorimetric method[19], CAT activity was determined via ultraviolet absorption method[20], while MDA content was determined using thiobarbituric acid (TBA) [21].

At the maturity, 20 plants of uniform growth were randomly selected from each treatment. Grains per row, weight of grains per panicle and 100-grain weight were determined after air drying. After threshing and weighing, the water content of grain was measured.

### Statistical analysis

Data were analyzed by one-way ANOVA, following a double-factor randomized block plot design. The significant differences among treatments were analyzed at 5% level of probability. SPSS 23.0 was used to perform all the statistical analyses.

## Results

### Chlorophyll fluorescence parameters

**Fig 5:**
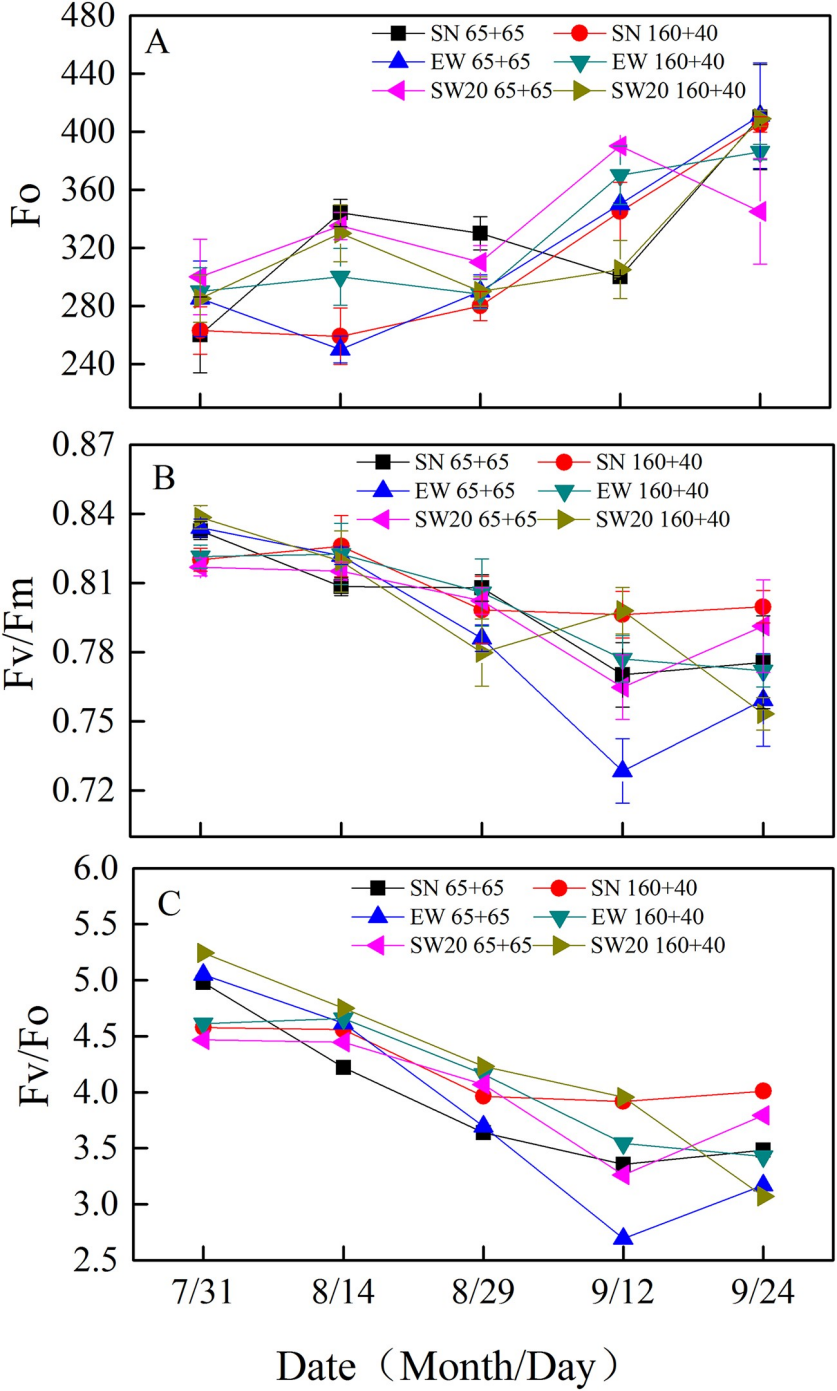
The changes of chlorophyll fluorescence parameters in ear leaf of maize after anthesis with different plating patterns in 2016. A, B and C represent the values of Fo, Fv/Fm and Fv/Fo respectively.

**Fig 6:**
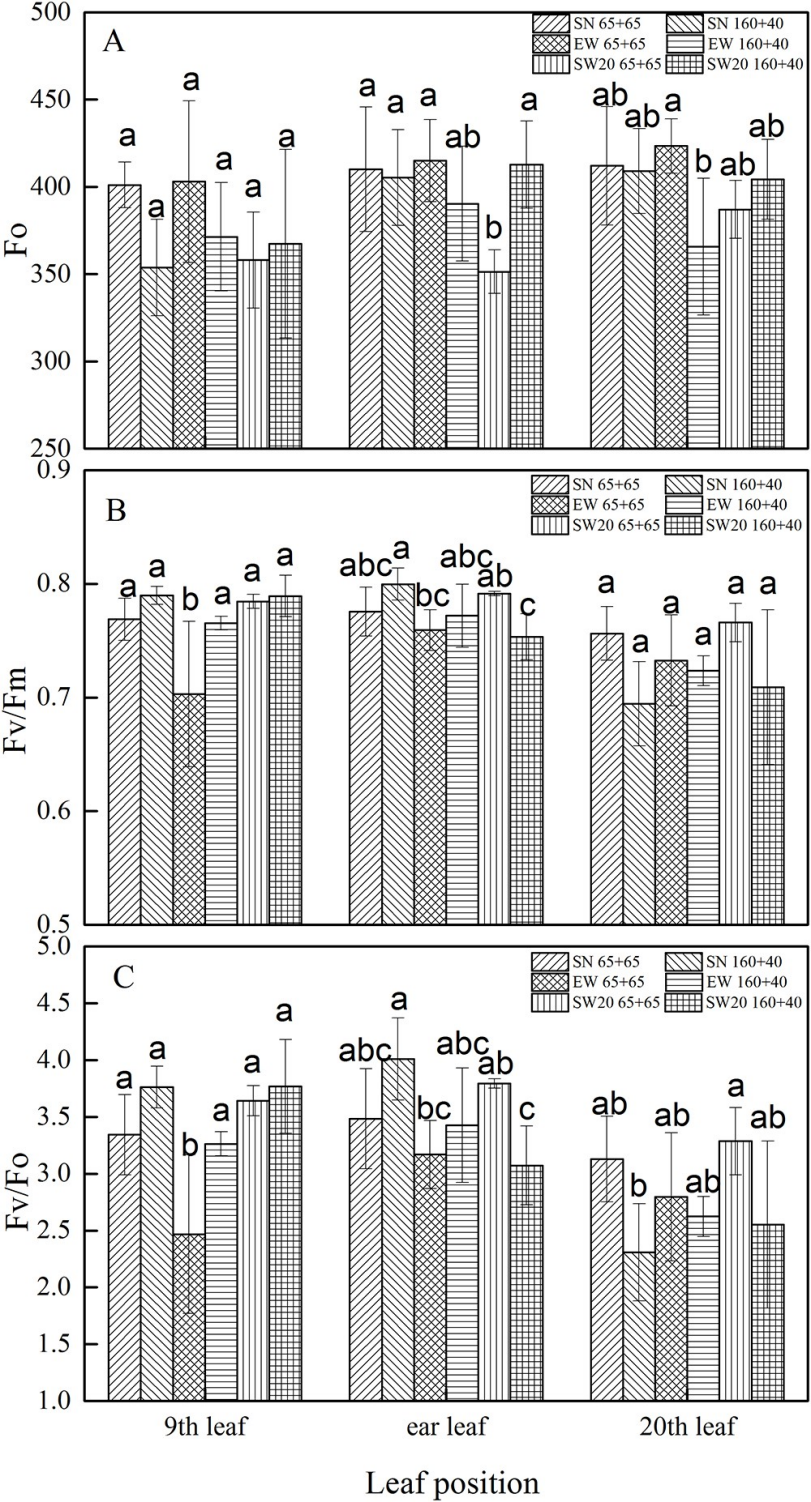
The Fo, Fv/Fm and Fv/Fo of maize leaves at different leaf positions during mature stage with different plating patterns in 2016.(showed as A, B and C in turn). Notes: SN: south-to-north orientation; EW: east-to-west orientation; SW20: northeast-to-southwest orientation, where the orientation was southwestern 20°; 65 + 65: 65 cm of both rows; 160 + 40: 40 cm of narrow row and 160 cm of wide row. Data are means ± SD (n = 5). Bars with different lower case letters indicate significant differences at P < 0.05.

The Φ_P0_ and Φ_N0_ of ear leaves decreased with planting patterns gradually (Fig 5 and S1 Table), while Fo showed an upward trend. Except for maturity stage, the Φ_N0_ of ear leaves under SW20 ‘160 + 40’ were higher than that under other planting patterns, possibly due to sampling errors and uneven fertilization. The Fo and Φ_P0_ were hardly different from other planting patterns. During maturity stage, the effect of different planting patterns on Fo, Φ_P0_ and Φ_N0_ of leaves in different parts of maize was shown in Fig 6 and S2 Table. The Φ_P0_ and Φ_N0_ of the upper leaves were slightly lower than those of the middle and lower leaves, while the Fo was opposite. In EW, the Φ_P0_ and Φ_N0_ of the lower leaves under ‘65 + 65’ were significantly lower than those under ‘160 + 40’ by 0.06 and 0.79, respectively. In SN and EW, the Fo under ‘65 + 65’ was higher than ‘160 + 40’. The Φ_P0_ and Φ_N0_ in the lower leaves under ‘160 + 40’ were higher than those under ‘65 + 65’. In ‘65 + 65’, the Fo of the middle leaves under SN and EW were significantly higher than that under SW20 by 80.91 and 67.51, respectively; the Φ_P0_ of the lower leaves under EW was significantly lower than that under SW20 by 0.07. The effect of directions on Φ_N0_ was not significant.

### Photosynthetic rate

**Fig 7:**
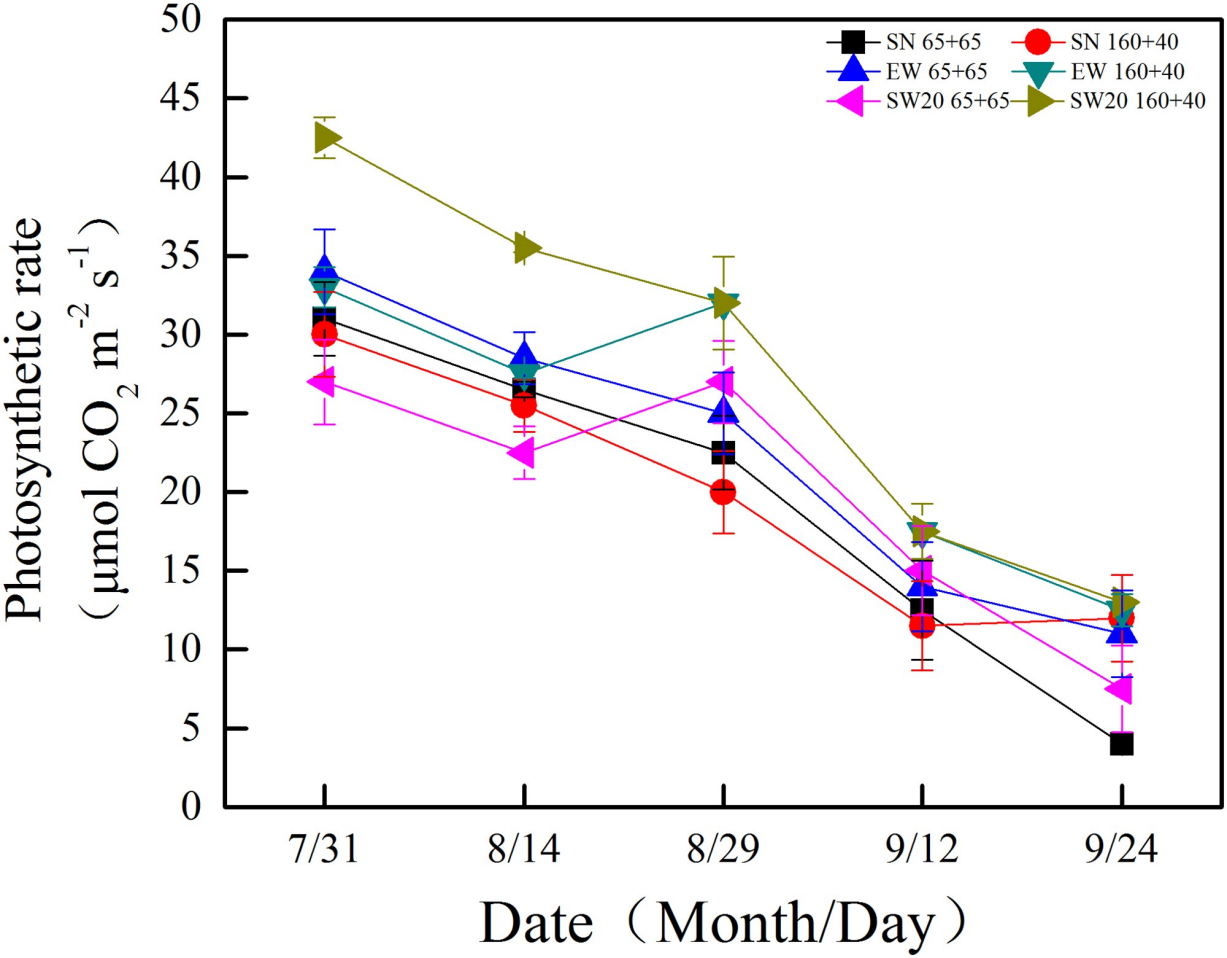
The changes of photosynthetic rate in ear leaf of maize after anthesis with different plating patterns in 2016. Notes: SN: south-to-north orientation; EW: east-to-west orientation; SW20: northeast-to-southwest orientation, where the orientation was southwestern 20°; 65 + 65: 65 cm of both rows; 160 + 40: 40 cm of narrow row and 160 cm of wide row.

Despite different planting patterns, the photosynthetic rate of ear leaf all decreased after anthesis (see Fig 7 and S1 Table). Among them, the photosynthetic rate of ear leaf under SW20 ‘160 + 40’ was higher than other planting patterns at five stages. During maturity stage, the effects of planting patterns on photosynthetic rate in different parts of maize were shown in Table 1. The photosynthesis rates of leaves of different parts decreased in the order: ear leaf > lower leaf> upper leaf. The photosynthesis rates at different directions decreased in the order: SW20> EW> SN. When we compared the photosynthesis rates under different row spacings, we could see that: in SN, the photosynthesis rates of the ear and lower leaves under ‘160 + 40’ were significantly higher than those under ‘65 + 65’ row spacing by 7.91μmol CO_2_ m^-2^ s^-1^ and 3.02μmol CO_2_ m^-2^ s^-1^, respectively; in EW, the photosynthesis rate of the upper leaves under ‘160 + 40’ was significantly higher than that under ‘65 + 65’ by 2.64μmol CO_2_ m^-2^ s^-1^; in SW 20, the leaves at different parts from top to bottom were significantly higher than that under ‘65 + 65’ by 4.44μmol CO_2_ m^-2^ s^-1^, 5.36μmol CO_2_ m^-2^ s^-1^ and 4.57μmol CO_2_ m^-2^ s^-1^, respectively. When we compared the photosynthesis rates under different directions, we could obtain that: in ‘65 + 65’, the photosynthesis rates of the lower leaves under SW20 were significantly higher than those under SN and EW by 1.67 μmol CO_2_ m^-2^ s^-1^ and 0.18μmol CO_2_ m^-2^ s^-1^, respectively; in ‘160 + 40’, the photosynthesis rates of the lower leaves under SW20 were significantly higher than those under SN and EW by 2.42μmol CO_2_ m^-2^ s^-1^ and 1.75μmol CO_2_ m^-2^ s^-1^. The photosynthesis rates under SW20 ‘160 + 40’ were 9.84μmol CO_2_ m^-2^ s^-1^, 12.60μmol CO_2_ m^-2^ s^-1^ and 7.56μmol CO_2_ m^-2^ s^-1^(from top leaves parts to bottom), which were the highest among all the treatments.

**Table 1:** The photosynthetic rate of maize leaves at different leaf positions during mature stage with different plating patterns in 2016(μmol CO_2_ m^-2^ s^-1^).

Row orientation	Row spacing	Leaf position		
		9^th^ leaf	Ear leaf	20^th^ leaf
SN	65 + 65	2.16±0.81d	3.90±0.12d	4.81±1.17b
	160 + 40	5.14±0.93b	11.71±1.62ab	2.08±0.12c
EW	65 + 65	3.54±0.30c	9.70±0.09bc	5.47±1.39b
	160 + 40	5.62±0.26b	12.12±0.94ab	7.91±1.95a
SW 20	65 + 65	3.73±0.35c	7.47±2.43c	5.40±0.98b
	160 + 40	7.56±0.09a	12.60±1.61a	9.84±0.52a

Notes: SN: south-to-north orientation; EW: east-to-west orientation; SW20: northeast-to-southwest orientation, where the orientation was southwestern 20°; 65 + 65: 65 cm of both rows; 160 + 40: 40 cm of narrow row and 160 cm of wide row. Data are means ± SD (n = 5). Lower case letters in the table indicate significant differences at P < 0.05.

### Protective enzyme activity and MDA content

**Fig 8:**
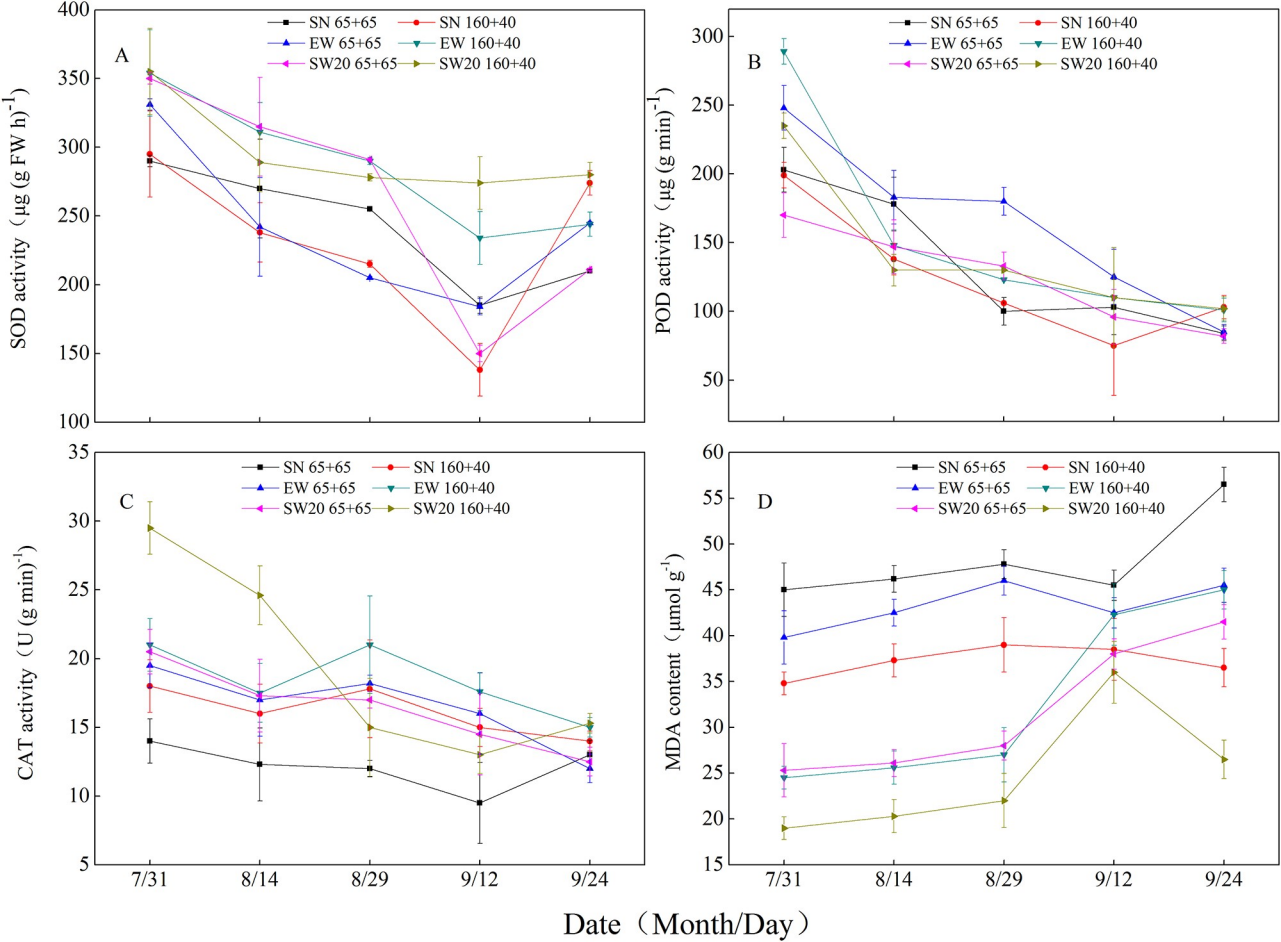
The changes of protective enzyme activity and MDA content in ear leaf of maize after anthesis with different plating patterns in 2017(showed as A, B,C and D in turn). Notes: SN: south-to-north orientation; EW: east-to-west orientation; SW20: northeast-to-southwest orientation, where the orientation was southwestern 20°; 65 + 65: 65 cm of both rows; 160 + 40: 40 cm of narrow row and 160 cm of wide row.

The SOD activity of ear leaves decreased gradually after anthesis and increased slightly after soft dough stage. POD and CAT activities continued to decrease, while MDA content increased. (see Fig 8 and S1 Table). The CAT activity of ear leaf under SW20 ‘160 + 40’ was higher in anthesis and milk stages and SOD activity was higher in milk-ripe, soft dough and maturity stages. However, POD content under SW20 ‘160 + 40’ was not significantly higher than other planting patterns. The MDA content of the ear leaf under SW20 ‘160 + 40’ was lower than other planting patterns at five stages.

**Fig 9:**
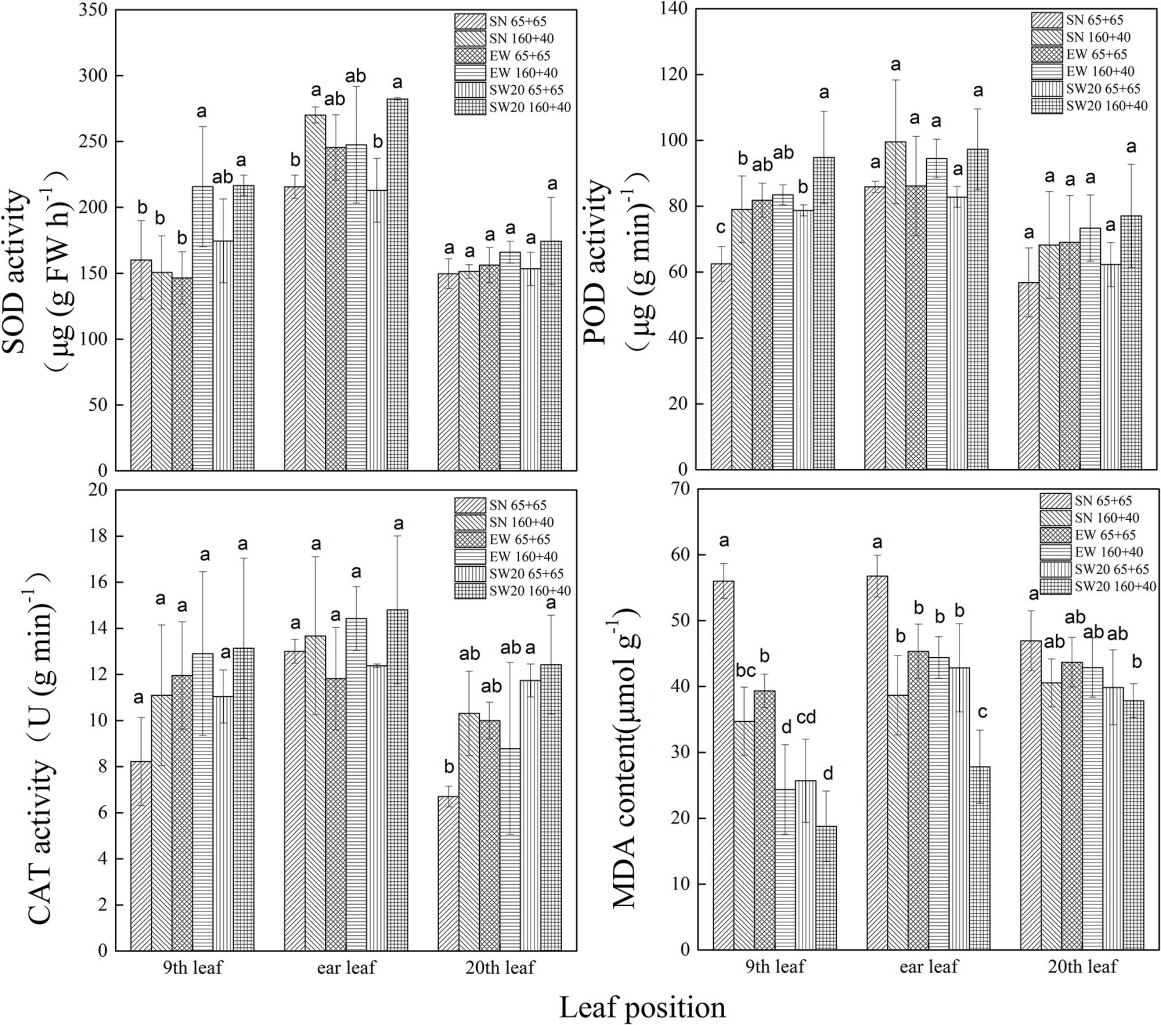
The protective enzyme activity and MDA content of maize leaves at different positions during mature stage with different plating patterns in 2017. (showed as A, B,C and D in turn). Notes: SN: south-to-north orientation; EW: east-to-west orientation; SW20: northeast-to-southwest orientation, where the orientation was southwestern 20°; 65 + 65: 65 cm of both rows; 160 + 40: 40 cm of narrow row and 160 cm of wide row. Data are means ± SD (n = 5). Bars with different lower case letters indicate significant differences at P < 0.05.

During maturity stage, the protective enzyme activities of maize leaves at different positions with different planting pattern were shown in Fig 9 and S2 Table. The protective enzyme activities of leaves at different positions were significantly different, and decreased in the order: ear leaves > lower leaf > upper leaf. The protective enzyme activities of leaves at different directions decreased in the order: SW20> EW> SN. For lower leaves, in EW, the SOD activity under ‘65 + 65’ was significantly lower than that under ‘160 + 40’ by 42.04 μg (g FW h)^-1^; in SN, the POD activity under ‘65 + 65’ was significantly lower than that under ‘160 + 40’by 17.51 μg (g min)^-1^; in SW20, the POD activity under ‘65 + 65’ was significantly lower than that under ‘160 + 40’by 16.85 μg (g min)^-1^; in ‘160 + 40’, the SOD activity under EW and SW20 were significantly higher than SN by 89.14 μg (g FW h)^-1^ and 97.10 μg (g FW h)^-1^, respectively; the POD activity under SW20 was significantly higher than that under SN by15.44 μg (g min)^-1^. For ear leaves, in SN, the SOD activity under ‘65 + 65’were significantly lower than that under ‘160 + 40’ by 31.09 μg (g FW h)^-1^; in SW20, the SOD activity under ‘65 + 65’ was significantly lower than that under ‘160 + 40’ by 40.91 μg (g FW h)^-1^; For the upper leaves, in ‘65 + 65’, the CAT activity under SW20 was significantly higher than that under SN by 4.81 U (g min)^-1^. For different positions leaves, the POD activity under ‘160 + 40’ was higher than ‘65 + 65’. Moreover, compared with other planting patterns, the SOD and CAT activity of leaves at different leaf positions under SW 20 ‘160 + 40’ was higher. During maturity stage, the effects of MDA content of maize leaves at different positions with different planting pattern were shown in Fig 6. It was shown that the upper and middle leaves were significantly higher than the lower leaves. The protective enzyme activity of leaves at different directions decreased in the order: SN > EW > SW20. The effect of different planting patterns on MDA content of the lower and ear leaves was significant. For the lower leaves, in SN, the MDA content under ‘65 + 65’ was significantly higher than that under ‘160 + 40’by 22.47 μmol g^-1^; in EW, the MDA content under ‘65 + 65’was significantly higher than that under ‘160 + 40’by 7.66 μmol g^-1^; in ‘160 + 40’, the MDA content under SW20 was significantly higher than those under SN and EW by 28.07μmol g^-1^ and 23.72μmol g^-1^ respectively. For the ear leaves in SN, the MDA content under ‘65 + 65’ was significantly higher than that under ‘160 + 40’ by 17.34 μmol g^-1^; in SW20, the MDA content of leaves under ‘65 + 65’ was significantly higher than that under ‘160 + 40’ by 17.98μmol g^-1^. In ‘65 + 65’, the MDA content under EW was significantly higher than that under SN and SW20 by 14.36μmol g^-1^ and 17.34 μmol g^-1^ respectively; in ‘160 + 40’, the MDA contents under SN and EW were significantly higher than that under SW20 by 13.27 μmol g^-1^ and 15.66 μmol g^-1^ respectively; The MDA content at different positions under ‘160 + 40’ was lower than that under ‘65 + 65’. Among all the planting patterns, SW20‘160 + 40’ led to the lowest MDA content at different leaf positions.

### Yield

The composition factors of production were shown in Table 2. The yield under different directions decreased in the order: SW20 > SN > EW. Redirected or not, the yield under ‘160 + 40’ was higher than that under ‘65 + 65’, by 7.07% in SN, by 7.68% in EW and 8.49% in SW20. In conclusion, SW20‘160 + 40’ had the highest yield of 11276.8 kg hm^-2^, which is higher than SN ‘65 + 65’, SN ‘160 + 40’, EW ‘65 + 65’ and EW‘160 + 40’ by 11.53%, 4.17%, 16.10% and 7.82%. The results showed that grains per row, 100-grain weight and kernel weight were positively correlated with yield.

**Table 2:** The grain yield components of maize in 2017.

Treatment	Denisity (plants m^−2^)	Line grain number	100-grain weight (g) 100-	Kernel weight (g)	Yield (kg ha^-1^)
SN 65 + 65	6.5	29.32±1.86cd	34.25±0.11cd	155.10±6.03d	10110.2±234.17e
SN 160 + 40	6.5	31.39±0.58ab	36.67±1.02ab	166.07±6.74b	10825.1±108.28b
EW 65 + 65	6.5	28.16±1.86d	32.90±0.26d	149.00±11.93d	9712.3±206.32f
EW 160 + 40	6.5	30.33±2.08bc	35.43±0.32bc	160.45±8.92c	10458.7±48.70c
SW 20 65 + 65	6.5	30.14±0.88bc	35.21±0.39bc	159.46±10.74c	10394.3±175.82d
SW 20 160 + 40	6.5	32.70±0.33a	38.20±1.23a	173.00±24.96a	11276.8±94.52a

Notes: SN: south-to-north orientation; EW: east-to-west orientation; SW20: northeast-to-southwest orientation, where the orientation was southwestern 20°; 65 + 65: 65 cm of both rows; 160 + 40: 40 cm of narrow row and 160 cm of wide row. Data are means ± SD (n = 5). Lower case letters in the table indicate significant differences at P < 0.05.

## Discussion

Maize is a high-light-efficiency C_4_ crop, whose canopy structure can be affected by the row configuration. Chlorophyll fluorescence is reddish-brown light, which is emitted by chlorophyll molecules with light stimulation under dark conditions. The fluorescence could be different depending on plant species and leaf age, which could predict carbon assimilation rate [22]. The Φ_P0_ and Φ_N0_ of the ear leaf under different planting patterns showed gradually decreasing trend [23]. The Φ_P0_ and Φ_N0_ of the upper leaves were lower, while the Fo was higher. Hence it’s confirmed that the upper leaves grew faster than lower ones.

Suitable distribution could form a good canopy structure and improve micro-environment, such as light, temperature, humidity and carbon dioxide content[24]. Our results showed that the photosynthetic rate of the ear leaf showed a downward trend after anthesis, indicating that photosynthesis decreased with leaf senescence. The photosynthetic rate of the ear leaves under SW 20 ‘160 + 40’ was significantly higher than other plating models. The photosynthesis rate at different leaf positions decreased in the order: middle leaves> upper leaves > lower leaves. It was due to the middle leaves had high leaf area index, and were at good ventilation and illumination conditions. The lower leaves deteriorate by light and result in decreasing photosynthetic performance[25]. The ear leaves had the highest photosynthesis in the late growth stage, providing good resources for grain filling.

Since Fridovich[18] proposed the bio-free radical hypothesis, broad attention has been drawn to plant stress resistance and aging mechanisms. Wang et al. [26] believed that high activity of protective enzymes and low membrane lipid peroxidation in the middle and lower leaves were favorable factors for high yield and stress resistance of maize varieties. The results showed that the POD and CAT activities in the ear leaves under different planting patterns decreased after anthesis, while the MDA content increased. The SOD activity decreased gradually after anthesis, but slightly increased after milk-ripe stage. These results were confirmed by Zhan et al. [27]. The reason might be POD and SOD played a synergistic role in the process of scavenging reactive oxygen species. In the late growth stage, the population density increased and the light transmission decreased, shortening the leaf function period and accelerating the aging process. When the SOD and POD contents in the cells were very low, the plant would increase SOD to resist the adverse environment[28]. After anthesis, the functional leaves of maize severely lost the ability of scavenging oxygen free radicals, so the peroxidation degree of cell membrane lipids was aggravated, leading to a decrease of the protective enzyme activity and a sharp increase of the MDA content[29]. The CAT activity of ear leaves under SW20 ‘160 + 40’ was higher in the anthesis and milk stages, while the SOD activity was higher during the milk-ripe, soft dough and maturity stages, indicating that SW20 ‘160 + 40’ delayed the senescence of the ear leaves by improving SOD in the early stage and CAT in the later stage, which was consistent with Wang et al. [30]. Compared to other planting patterns, the protective enzyme activity was higher and the MDA content was lower in SW20 ‘160 + 40’. In SW20 ‘160 + 40’, the damage of cell enzyme and membrane system was lower than other planting patterns, which helped slow down the leaf senescence. [31]. The POD activity in the leaves under ‘160 + 40’ was higher than that under ‘65 + 65’, while the MDA content was lower. Compared with the traditional planting patterns, the light conditions and the photosynthesis of the functional leaves in wide and narrow planting patterns were improved, ‘160 + 40’ had a strong ability to scavenge reactive oxygen and superoxide anion radicals which was crucial for delaying scenescence. The leaf senescence under ‘65 + 65’could be also a result of limited soil water availability caused by higher canopy rainfall interception[32].

Recently, the practice of maize production showed that the increase of yield mainly relied on cultivation techniques and increasing yield potential. To get high yields, the population structure should be reasonable and the leaves should maintain a high photosynthetic rate. In this study, we implemented filed experiments with multiple planting patterns, the results showed that the yield under ‘160 + 40’ was higher than that under ‘65 + 65’, indicating that reasonable row spacing significantly could improve the ventilation and light transmission conditions in fields [33], and further promote the growth and development. The yield under different directions decreased in the order: SW20 > SN > EW, which was consistent with Wang et al. [34]. The yield of maize under SW20 ‘160 + 40’ was significantly higher than that other planting patterns.

For Dehui fields, SW20 could also shorten the horizontal projection of the planting line so that the plants could receive the long-time illumination. The row spacing was further increased to 160 cm. If we assumed that the mutual shading was the smallest and the light energy utilization was the best, then ventilation and light conditions could be further improved [35]. We concluded that SW20 ‘160 + 40’ should be carried out in Dehui City, Jilin Province.

## Conclusion

In this paper, we experimentally demonstrated that SW 20 ‘160 + 40’ was the most favorable choice among all the planting patterns. SW 20 ‘160 + 40’ could help increase the photosynthetic rate of ear leaf; during the maturity stage, SW 20 ‘160 + 40’ could help remove the active oxygen free radicals in different parts of maize; in addition, SW 20 ‘160 + 40’ could also help prevent cells from damage, maintain the integrity of the structure, delay leaf senescence and increase maize yield. Therefore, in De Hui field site, SW 20 ‘160 + 40’ planting pattern significantly delayed leaf senescence in the late maize growth, and intensely increased the maize yield.

## Supporting information

S1 Table The changes of chlorophyll fluorescence parameters, photosynthetic rate, protective enzyme activity and MDA content at different times. (XLSX)

S2 Table The chlorophyll fluorescence parameters, protective enzyme activity and MDA content of maize leaves at different positions. (XLSX)
